# Botulinum Toxin's Effects on Muscle Tone and Joint Mobility in Children with Congenital Zika Syndrome – A Case Series

**DOI:** 10.1055/s-0044-1792114

**Published:** 2024-12-21

**Authors:** Patrícia Juliana da Silva, Ana Stela Salvino de Brito, Mariana Balbino da Silva, Nathalia Nogueira Romariz Barros, Jousilene Sales Tavares, Gabriela Lopes Gama

**Affiliations:** 1 Instituto de Pesquisa Professor Joaquim Amorim Neto (IPESQ), Campina Grande, PB, Brasil; 2 Universidade Federal de Juiz de Fora (UFJF), Governador Valadares, MG, Brasil

**Keywords:** arthrometry articular, muscle hypertonia, muscle spasticity, Zika virus infection

## Abstract

**Objective** To evaluate the effects of the botulinum toxin (BTX-A) on muscle tone and joint mobility in children with congenital Zika syndrome (CZS).

**Methods** This was a longitudinal case series carried out in a Support Center for Children with Microcephaly, located in Northeastern Brazil. We collected data from the institution's medical records, containing information about muscle tone and passive joint mobility measured at least 3 months before and 4 weeks after BTX-A application.

**Results** We evaluated 13 children (9 boys) with a mean age of 77 ± 7.1 months. After BTX-A application, a bilateral reduction in the hypertonia level was observed in the elbow flexor ( *p* < 0.01) and hip abductor ( *p* < 0.05) muscles.

**Conclusion** No changes were observed in joint mobility and no adverse effects were reported by caregivers after application. The use of BTX-A can reduce hypertonia in CZS children, with no impact on joint mobility.

## Introduction

First described in 2015, the congenital Zika syndrome (CZS) is characterized by brain malformations resulting from intrauterine infections by the Zika virus. 1 At birth, the main clinical finding in children is microcephaly. However, other signs become evident during development, including delayed motor skill acquisition, difficult-to-control seizures, changes in muscle tone, and others. 2 3 4

With special regards to the muscle tone in children with CZS, Pereira et al. 4 described clinical signs of pyramidal lesions, such as hypertonia and hyperreflexia, in 93% of the subjects evaluated. Corroborating these findings, Tavares et al. 2 observed the presence of appendicular hypertonia in 94.8% of the children from their sample. These findings, which may result from musculoskeletal hypertonia and daily care, 5 6 suggest the need for therapeutic approaches to treat muscle tone changes in this population.

In this context, the intramuscular application of botulinum toxin type-A (BTX-A) has been described as an efficient therapeutic approach for controlling hypertonia in patients with neurological conditions. 7 8 9 This application aims at the reversible chemical denervation of the muscle with an increased tone, reducing inappropriate muscle activity. 10

In children with cerebral palsy (CP), studies have shown positive outcomes after BTX-A application, in muscle tone control and improving joint mobility and functionality. 7 11 12 In children with CZS, the procedure showed positive effects for sialorrhea control. 13 Moreover, in this population, the only study to investigate the BTX-A effects on the musculoskeletal system described hypertonia reduction and joint mobility improvement based on parental reports. 14 However, this last study did not describe the effects of this therapeutic approach on the hypertonia level of specific muscle groups and the range of joint motion.

Considering the promising results of BTX-A and the need for a better understanding of its impact in children with CZS, our study aims to evaluate its effects on muscle tone and joint mobility in this population.

## Materials and Methods

The present is a longitudinal case series conducted at Dr. Arthur Eugênio Azevedo Support Center for Children with Microcephaly from the Professor Joaquim Amorim Neto Research Institute (IPESQ), in Campina Grande, Paraíba, Brazil. The Ethics and Research Committee of the Higher Education and Development Center approved this study (CAAE: 58018022.9.0000.5178). Before data collection, the person in charge of the support center authorized file handling.

### Sample

Sample recruitment was nonprobabilistic, occurring conveniently among children attending the support center mentioned above. The inclusion criteria were (1) having a CZS diagnosis confirmed by reverse transcription polymerase chain reaction (RT-PCR), or imaging exams performed in the first months of life; (2) presenting increased muscle tone in at least one group with a grade higher than 1 per the modified Ashworth scale (MAS); and (3) undergoing BTX-A application at the study's center. We excluded children with fixed contractures, not evaluated by professionals from our specialized center at least 3 months before or 4 weeks after the BTX-A application, and those receiving the drug 6 months before the first evaluation.

### Data Collection Procedures

We collected data from clinical records from the Dr. Arthur Eugênio Azevedo Support Center for Children with Microcephaly. The children who met the study eligibility criteria received BTX-A at this center and were included in this study. We extracted general data, such as weight, head circumference, and presence of microcephaly at birth, from their medical records, in addition to their age, weight, head circumference at the time of drug treatment, and level of motor impairment according to the Gross Motor Function Classification Measure (GMFCS). We also recorded information regarding BTX-A administration, including the date and the muscles for medication injection.

The assessment of BTX-A effects relied on data from up to 3 months before (preassessment) and 4 weeks after (postassessment) its administration. These data included muscle tone evaluation by MAS, 15 and passive joint mobility determined by goniometric measurements. 16

We used MAS to measure muscle resistance to passive movement and assesses muscle tone in patients with neurological impairments, such as children with CZS 2 and CP. 7 According to this scale, resistance to passive motion ranges from 0 (no increase in muscle tension) to 4 (rigid segment with no movement). 17 In our study, we assessed the following muscle groups of the upper limbs using MAS: shoulder flexors and adductors, elbow extensors and flexors, wrist extensors and flexors, and finger flexors. In the lower limbs, the muscle groups assessed were the hip flexors, hip adductors, knee flexors and extensors, dorsiflexors, and plantar flexors.

We measured the maximum passive joint range of motion using a manual goniometer. This assessment considered shoulder, elbow, wrist, hip, knee, and ankle joint movements in the sagittal plane, as well as shoulder and hip joint abduction movements in the frontal plane.

All children received BTX-A at the outpatient clinic from the Dr. Arthur Eugênio Azevedo Support Center for Children with Microcephaly after use of 20 mg lidocaine hydrochloride gel in the application regions. The same professional performed all procedures, considering the hypertonia level and the needs of each child. The procedure did not require any auxiliary instrument. Additionally, trained professionals with experience in treating children with CZS and using the assessment instruments conducted all evaluations before and after the BTX-A application.

### Statistical Analysis

Descriptive statistics characterize the sample, considering mean and standard deviation values for continuous variables, such as the child's age and maximum passive joint range assessed before and after the application of BTX-A. We calculated the absolute and relative frequencies of categorical variables, such as the MAS scores before and after the procedure.

Then, we proceeded to inferential statistics. The paired Wilcoxon tests were used, considering MAS scores as dependent variables to evaluate the procedure's effects on muscular resistance to passive movement. To evaluate BTX-A on joint mobility, we initially calculated data normality using the Shapiro-Wilk test. Since data distribution was non-normal, the nonparametric paired Wilcoxon test compared the variables. We did all analyses using the Medcalc (MedCalc Software BVBA, Ostend, Belgium), version 19.0.7, and the statistical significance level was 5%.

## Results

The total sample consisted of 13 children (9 boys), of whom 7 (53.8%) had microcephaly at birth. At the time of BTX-A administration, the children were aged 70 to 89 (77 ± 7.1) months. None of them could walk independently, 12 (92.3%) had spastic tetraparesis, and all presented severe motor impairment, classified as levels IV (15.4%) and V (84.6%) per GMFCS. Table 1 shows the general individual data of the children participating in the study.

**Table 1:** Individual profile of the children evaluated and muscle groups of BTX-A injection

Patient	Age at application (months)	Gender	Head circumference (cm)		Weight (kg)		Muscle groups for BTX-A application
			At birth	At application	At birth	At application	
1	71	M	32.5	45	3.32	16.5	Biceps brachii, wrist flexors, finger flexors, and triceps surae
2	77	M	32	45	3.3	19.02	Biceps brachii, wrist flexors, finger flexors, and hamstrings
3	77	M	33	49	3.06	23.24	Biceps brachii, wrist flexors, hip adductors, hamstrings, and triceps surae
4	70	F	28	41.5	3.01	17.83	Hip adductors and triceps surae
5	70	F	31.2	45.5	3.55	20.63	Biceps brachii, finger flexors, hamstring, and triceps surae
6	76	M	31	49.5	2.02	22.86	Hip adductors and triceps surae
7	78	M	30	40	2.95	11.21	Biceps brachii, wrist flexors, hip adductors, and hamstrings
8	87	M	30	45	2.78	17.94	Hip adductors, hamstring, and triceps surae
9*	76	F		41		16.59	Biceps brachii, finger flexors, hip adductors, and hamstrings
10	77	M	29	38.5	2.60	13.60	Wrist flexors and hip adductors
11	89	F	29.9	37.5	3.31	14.02	Hip adductors and hamstrings
12	88	M	29	45	2.64	17.83	Biceps brachii, wrist flexors, and hamstrings
13	89	M	32	47	2.76	24.86	Finger flexors, hip adductors, and triceps surae

**Abbreviations:** BTX-A, botulinum toxin-A; F, female; M, male. **Notes:** * Adopted child with no birth information.

The BTX-A was administered to the upper limbs of 9 children, especially at the biceps brachii (n = 7) and the flexor carpi ulnaris muscle (n = 6). In the lower limbs, the application occurred in all children in at least one muscle group, mostly hip adductors (n = 9), hamstrings (n = 8), and triceps surae (n = 7). Caregivers reported no adverse effects between assessments.

After application, no changes were observed in the maximum passive joint range for any movement evaluated ( Table 2 ). In contrast, regarding muscle tone, we noted significant differences in the hypertonia degree on the right ( *p* = 0.01) and left ( *p* = 0.008) elbow flexor muscle groups, and right ( *p* = 0.02) and left ( *p* = 0.04) hip abductors, as shown in Table 3 .

**Table 2:** Joint range of movement before and after BTX-A application

Joint movement	Preapplication			Postapplication			
	Median	Min	Max	Median	Min	Max	*p* -value
**Shoulder flexion**							
Right	170	90	180	168	90	180	0.93
Left	156	90	180	150	90	180	0.68
**Shoulder elevation**							
Right ^a^	45	45	45	45	45	45	–
Left ^a^	45	45	45	45	45	45	–
**Shoulder abduction**							
Right	160	90	180	150	110	180	0.68
Left	160	90	180	140	110	180	0.84
**Elbow flexion**							
Right ^a^	145	145	145	145	145	145	–
Left ^a^	145	145	145	145	145	145	–
**Elbow extension**							
Right	0	0	120	0	0	60	0.12
Left	0	0	66	0	0	70	0.62
**Wrist flexion**							
Right ^a^	90	30	140	90	30	70	–
Left	90	0	140	90	20	70	1.00
**Wrist extension**							
Right ^a^	70	0	70	70	20	70	–
Left	70	20	70	70	20	70	0.81
**Hip flexion**							
Right	110	90	125	125	90	125	0.29
Left	110	90	125	120	90	125	0.62
**Hip extension**							
Right	10	0	10	10	0	10	0.62
Left	10	0	10	10	0	10	0.31
**Hip abduction**							
Right	30	2	45	35	6	45	0.46
Left	24	0	45	30	8	45	–
**Hip adduction**							
Right ^a^	15	8	15	15	15	15	–
Left ^a^	15	15	15	15	15	15	–
**Knee flexion**							
Right ^a^	140	140	140	140	140	140	–
Left ^a^	140	140	140	140	140	140	–
**Knee extension**							
Right	0	0	28	0	0	30	1.00
Left	10	0	135	0	0	40	0.06
**Dorsiflexion**							
Right	20	10	30	0	0	20	0.81
Left ^a^	20	10	20	20	0	20	–
**Plantar flexion**							
Right	45	30	45	45	35	45	0.21
Left	45	30	45	45	25	45	0.87

**Abbreviation:** BTX-A, botulinum toxin-A.

**Note:**^a^ Statistical tests were not possible as there were no numerical changes in the joint angle between assessments.

## Discussion

Our findings demonstrated that the BTX-A application can promote a tone graduation in children with CZS, despite not affecting joint mobility. Furthermore, we recorded no adverse effects 4 weeks after the application.

Spastic hypertonia characterizes the muscle tone of most children with CP 18 and CZS, 2 often resulting in reduced mobility, contractures, and deformities potentially compromising development and functionality. 19 Specifically in children with reduced mobility, such as those with GMFCS levels IV and V, generalized hypertonia also results in pain, discomfort, and difficulties in daily care. 20 21 Thus, for this population, simple tasks, such as changing clothes, can be difficult.

Despite the impacts of increased muscle tone on the health and quality of life of children with severe motor impairments who are unable to walk, studies evaluating the effects of BTX-A are scarce. This can be explained by the need to apply it to multiple muscles and a higher risk of adverse effects. 7 On the other hand, a systematic review by Pin et al. 22 showed that BTX-A applied to children with GMFCS levels IV and V was efficient in reducing pain, facilitating daily activities, and promoting motor skill improvements. However, these outcomes should be viewed with caution since most studies had low or moderate methodological quality. 22

In children with CZS, only two of the studies investigated the effects of BTX-A use. One of them evaluated its impacts in the sialorrhea treatment, 13 demonstrating an improvement in symptom severity and the occurrence of adverse effects in a small portion of the sample (2/23). The other study investigated the effects of BTX-A on spasticity and motor performance in a sample predominantly presenting severe motor impairment (85%). Their results demonstrated that most parents reported improvements in their children's range of motion or spasticity after the application of BTX-A, without any adverse effects. 14

Despite the importance of these results, previous studies did not demonstrate the impacts of BTX-A application on the muscle tone of specific muscle groups evaluated using scales such as MAS. This occurred because Armani-Franceschi et al. 14 evaluated only the sum of MAS scores, rather than the individual muscle groups evaluated, which limited the detailed understanding of the impacts of BTX-A in children with CZS.

Tavares et al. 2 proposed the evaluation of hypertonia of specific muscle groups by MAS in a cross-sectional study in which most children with CZS presented axial hypotonia and appendicular hypertonia, with the elbow flexors and hip adductors being more resistant to passive movement. These muscle groups were among those most frequent receiving BTX-A in the present studies (53.8 and 69.2%, respectively), unlike the study by Armani-Franceschi et al., 14 in which 50% of the children received the drug in the long adductor and 35% in the biceps brachii.

In children with neurological impairments, hypertonia of the hip muscles is common, resulting in a higher susceptibility to dislocations and pain. 19 Specifically in children with CZS, a high prevalence of hip dislocation and sublocation has been described, apparently related to the hypertonia level, 23 resulting in pain and functional limitations. 24 Given these findings, the reduced resistance to passive movement after the BTX-A application observed by us may represent a therapeutic alternative for preserving the hips of these children, relieving pain, and facilitating their daily care.

To date, no study has evaluated the impacts of hypertonia in children with CZS. Despite this, the reduction in resistance to passive movement of the elbow flexors described by our study may represent a positive point, facilitating daily care, play, and the child's interaction with the environment. This can occur because children with severe motor impairments present upper limb muscle tone grades, facilitating reaching tasks and playing with toys. 7

We observed no statistical differences regarding joint mobility based on the maximum passive joint range of motion. This finding may raise some hypotheses. First is the multicausal nature of reduced joint range of motion, including hypertonia and other factors, such as muscle and tendon shortening, extracellular matrix abnormalities, and others, that can compromise joint mobility in children with neurological impairment. 25 26 27 The second hypothesis is the need for a longer association between BTX-A and physical therapy programs to achieve significant therapeutic outcomes. 28

Despite the absence of differences in joint range of motion before and after BTX-A application, during the study period, physical therapists monitored the children evaluated and reported that their motor skills were better during the sessions after the procedure. This observation may result from the reduced resistance to passive movement in adjacent body segments. Although this finding was not an outcome of the present study, it should be considered since facilitating handling in rehabilitation programs can lead to better long-term therapeutic responses.

It is worth highlighting that our results were potentially influenced by monitoring of the children by a multidisciplinary team, including physical therapists. However, we did not control the number of sessions and interventions performed, which can be a limitation of our study. Other limitations deserve consideration, such as the small number of well-evaluated children, the variety of muscle groups for BTX-A application, and the short follow-up period after the procedure. Therefore, we suggest that future studies involve a larger number of participants with serial and longer-term follow-ups to verify the prolonged effects of this application.

## Conclusion

Despite the generalized hypertonia presented by children with CZS, our study only performed the application of BTX-A in some muscle groups, defined individually and based on the clinical evaluation of a specialized multidisciplinary team to avoid overdose. This may explain the absence of adverse effects in the participating children and suggests the need for a specialized and experienced clinical approach for this population.

Treatment with BTX-A can promote muscle tone graduation in children with CZS, reducing the resistance to passive movement. Nevertheless, this therapy could not change joint mobility, as it is determined by the passive range of motion.

**Table 3:** Assessment of the spasticity level according to the MAS before and after botulinum toxin-A application

Muscle group assessed	MAS preapplication, N (%)						MAS postapplication, N (%)						
	0	1	1+	2	3	4	0	1	1+	2	3	4	*p* -valor
**Shoulder adductors**													
Right	5 (38.5)	1 (7.7)	2 (15.4)	3 (23.1)	2 (15.4)	0	4 (30.8)	6 (46.2)	0	3 (23.1)	0	0	0.15
Left	6 (46.2)	2 (15.4)	1 (7.7)	2 (15.4)	2 (15.4)	0	3 (23.1)	8 (61.5)	0	2 (15.4)	0	0	0.54
**Hip flexors**													
Right	7 (53.8)	3 (23.1)	0	3 (23.1)	0	0	9 (69.2)	4 (30.8)	0	0	0	0	0.09
Left ^a^	7 (53.8)	3 (23.1)	0	3 (23.1)	0	0	8 (61.5)	3 (23.1)	0	2 (15.)	0	0	–
**Shoulder flexors**													
Right	5 (38.5)	2 (15.4)	2 (15.4)	1 (7.7)	3 (23.1)	0	5 (38.5)	5 (38.5)	1 (7.7)	1 (7.7)	1 (7.7)	0	0.56
Left	4 (30.8)	4 (30.8)	1 (7.7)	1 (7.7)	3 (23.1)	0	3 (23.1)	6 (46.2)	1 (7.7)	2 (15.4)	1 (7.7)	0	0.08
**Elbow extensors**													
Right	9 (75)	2 (16.7)	0	0	1 (8.3)	0	8 (61.5)	3 (23.1)	1 (7.7)	1 (7.7)	0	0	1.00
Left	8 (66.7)	3 (25)	0	0	1 (8.3)	0	9 (69.2)	4 (30.8)	0	0	0	0	0.62
**Elbow flexors**													
Right	2 (15.4)	3 (23.1)	1 (7.7)	4 (30.8)	1 (7.7)	2 (15.4)	6 (46.2)	3 (23.1)	0	2 (15.4)	1 (7.7)	1 (7.7)	0.01
Left	3 (23.1)	2 (154)	1 (7.7)	4 (30.8)	1 (7.7)	2 (15.4)	7 (53.8)	3 (23.1)	0	1 (7.7)	1 (7.7)	1 (7.7)	0.008
**Wrist extensors**													
Right ^a^	8 (61.5)	3 (23.1)	1 (7.7)	1 (7.7)	0	0	10 (76.9)	2 (15.4)	0	1 (7.7)	0	0	–
Left	8 (61.5)	3 (23.1)	0	2 (15.4)	0	0	10 (76.9)	2 (15.4)	0	0	1 (7.7)	0	0.87
**Wrist flexors**													
Right	8 (61.5)	1 (7.7)	1 (7.7)	0	2 (15.4)	1 (7.7)	8 (61.5)	1 (7.7)	0	1 (7.7)	3 (23.1)	0	1.00
Left	9 (69.2)	1 (7.7)	1 (7.7)	0	1 (7.7)	1 (7.7)	10 (76.9)	1 (7.7)	0	1 (7.7)	1 (7.7)	0	0.56
**Finger flexors**													
Right ^a^	9 (69.2)	2 (15.4)	0	1 (7.7)	1 (7.7)	0	10 (76.9)	2(15.4)	0	0	1 (7.7)	0	–
Left	10 (76.9)	1 (7.7)	0	2 (15.4)	0	0	9 (69.2)	3 (23.1)	1 (7.7)	0	0	0	0.68
**Hip adductors**													
Right	2 (15.4)	2 (15.4)	1 (7.7)	5 (38.5)	3 (23.1)	0	3 (23.1)	5(38.5)	2 (15.4)	3 (23.1)	0	0	0.02
Left	2 (15.4)	2 (15.4)	1 (7.7)	5 (38.)	3 (23.1)	0	4 (30.8)	3 (23.1)	2 (15.4)	4 (30.8)	0	0	0.04
**Knee extensors**													
Right	9 (69.2)	1 (7.7)	0	3 (23.1)	0	0	7 (53.8)	3 (23.1)	2 (15.4)	1 (7.7)	0	0	1.00
Left	9 (69.2)	1 (7.7)	0	3 (23.1)	0	0	6 (46.2)	4 (30.8)	2 (15.4)	1 (7.7)	0	0	0.91
**Knee flexors**													
Right ^a^	10 (76.9)	2 (15.4)	0	1 (7.7)	0	0	11 (84.6)	1 (7.7)	1 (7.7)	0	0	0	–
Left	9 (69.2)	2 (15.4)	1 (7.7)	1 (7.7)	0	0	12 (92.3)	0	0	1 (7.7)	0	0	0.37
**Plantar flexors**													
Right	2 (15.4)	5 (38.5)	2 (15.4)	2 (15.4)	2 (15.4)	0	4 (30.8)	2 (15.4)	0	5 (38.5)	0	2 (15.4)	0.82
Left	1 (7.7)	5 (38.5)	3 (23.1)	2 (15.4)	2 (15.4)	0	3 (23.1)	1 (7.7)	0	7 (53.8)	2 (15.4)	0	0.49
**Dorsiflexors**													
Right	6 (46.2)	3 (23.1)	2 (15.4)	0	2 (15.4)	0	8 (61.5)	3 (23.1)	0	1 (7.7)	0	1 (7.7)	0.64
Left	6 (46.2)	3 (23.1)	2 (15.4)	0	2 (15.4)	0	9 (69.2)	1 (7.7)	0	3 (23.1)	0	0	0.57

**Abbreviations:** MAS, modified Ahworth scale.

**Note:**^a^ Statistical tests were not possible as there were no numerical changes in the joint angle between assessments.
