# Supplementary material for: Botulinum Toxin's Effects on Muscle Tone and Joint Mobility in Children with Congenital Zika Syndrome – A Case Series
Source: Rev Bras Ortop (Sao Paulo). 2024 Dec 21;59(6):e936–43. doi: 10.1055/s-0044-1792114 (PMC11663055; doi:10.1055/s-0044-1792114)
Supplement: Related texts

# Research article: Efeitos da toxina botulínica sobre o tônus muscular e a mobilidade articular de crianças com síndrome congênita do Zika – Uma série de casos

**Keywords:** artrometria articular, espasticidade muscular, hipertonia muscular, infecção pelo Zika vírus

## Abstract

**Objetivo** Avaliar os efeitos da toxina botulínica **(** BTX-A) sobre o tônus muscular e mobilidade articular de crianças com síndrome congênita do Zika (SCZ).

**Métodos** Trata-se de uma série de casos longitudinal realizada em um Centro de Apoio a Criança com Microcefalia, localizado no nordeste do Brasil. A coleta de dados foi realizada a partir dos prontuários dessa instituição, com o registro de informações sobre o tônus muscular e a mobilidade articular passiva, mensuradas pelo menos 3 meses antes e 4 semanas após a aplicação da BTX-A.

**Resultados** Foram avaliadas 13 crianças (9 meninos) com idade média de 77 ± 7,1 meses. Após a aplicação da BTX-A, foi observada redução bilateral do nível de hipertônia nos músculos flexores do cotovelo ( *p* < 0,01) e adutores de quadril ( *p* < 0,05) bilateralmente.

**Conclusão** Nenhuma mudança foi observada na mobilidade articular e nenhum efeito adverso foi relatado pelos cuidadores após a aplicação. O uso da BTX-A é capaz de promover a redução do nível de hipertonia de crianças com SCZ, sem impactar a mobilidade articular.

## Introdução

Descrita pela primeira vez em 2015, a síndrome congênita do Zika (SCZ) é caracterizada por malformações encefálicas resultantes de infecções intraútero pelo Zika vírus. 1 Ao nascimento, o principal achado clínico de crianças com esse diagnóstico é a microcefalia. Entretanto, ao longo do desenvolvimento, outros sinais tornam-se evidentes como atraso na aquisição de habilidades motoras, crises convulsivas de difícil controle, alterações no tônus muscular dentre outras. 2 3 4

Especificamente no que se refere ao tônus muscular de crianças com SCZ, Pereira et al. 4 descreveram a presença de sinais clínicos de lesões piramidais como a hipertônia e hiperreflexia em 93% das crianças avaliadas. Corroborando esses achados, Tavares et al. 2 observaram a presença de hipertonia apendicular em 94.8% das crianças de sua amostra. Esses achados, atrelados às possíveis consequências da hipertônia sobre o sistema musculoesquelético e cuidados cotidianos, 5 6 sugerem a necessidade de abordagens terapêuticas voltadas para o tratamento das alterações no tônus muscular dessa população.

Nesse contexto, a aplicação intramuscular da toxina botulínica tipo-A (BTX-A) tem sido descrita como uma abordagem terapêutica eficiente para o controle da hipertônia em pacientes com distúrbios neurológicos. 7 8 9 Essa aplicação visa a desenervação química reversível do músculo que apresenta aumento do tônus muscular, tendo como consequência a redução da atividade muscular inadequada. 10

Em crianças com paralisia cerebral (PC), estudos têm mostrado resultados positivos quanto a aplicação de BTX-A, não apenas para o controle do tônus muscular, mas também para melhora da mobilidade articular e funcionalidade. 7 11 12 Em crianças com SCZ, o procedimento apresentou efeitos positivos para controle da sialorreia. 13 Ainda nessa população, o único estudo a investigar os efeitos da BTX-A sobre o sistema musculoesquelético descreveu a redução da hipertonia e melhora da mobilidade articular a partir de relato dos pais. 14 Esse último estudo, entretanto, não descreveu os efeitos dessa abordagem terapêutica sobre o nível de hipertonia de grupos musculares específicos e da amplitude de movimento articular.

Considerando os resultados promissores do uso da BTX-A e a necessidade de uma melhor compreensão dos impactos dessa abordagem terapêutica em crianças com SCZ, o presente estudo tem como objetivo avaliar seus efeitos sobre o tônus muscular e mobilidade articular nessa população.

## Materiais e Métodos

Trata-se de uma série de casos de caráter longitudinal realizado no Centro de Apoio a Criança com Microcefalia Dr. Arthur Eugênio Azevedo ligado ao Instituto de Pesquisa Professor Joaquim Amorim Neto (IPESQ), localizado na cidade de Campina Grande. O estudo foi aprovado pelo Comitê de Ética e Pesquisa do centro de ensino superior e desenvolvimento (CAAE: 58018022.9.0000.5178). Antes da coleta de dados, o responsável do centro de apoio autorizou o manuseio de seus arquivos.

## Amostra

A amostra foi recrutada de forma não probabilística por conveniência entre as crianças que frequentavam o centro de apoio citado anteriormente. Foram considerados como critérios de inclusão (1) ter diagnóstico de SCZ confirmado através da reação de transcriptase reversa seguida de reação em cadeia da polimerase (RT-PCR), ou exames de imagem realizados nos primeiros meses de vida; (2) apresentar aumento do tônus muscular em pelo menos um grupo, sendo este classificado acima de 1 de acordo com a escala de Ashworth modificada (EAM); e (3) ter aplicado BTX-A no centro especializado onde o estudo foi realizado. Foram excluídas crianças com contraturas fixas, aquelas que não foram avaliadas por profissionais do centro especializado onde o estudo foi realizado pelo menos 3 meses antes e 4 semanas após a aplicação da BTX-A e (4) aqueles que receberam o tratamento 6 meses antes da primeira avaliação.

## Procedimentos de Coleta de Dados

A coleta de dados foi realizada a partir dos registros clínicos do Centro de Apoio a Criança com Microcefalia Dr. Arthur Eugênio Azevedo. Foram selecionadas crianças que realizaram a aplicação de BTX-A nesse centro e se enquadraram nos critérios de elegibilidade do estudo. A partir dos prontuários das crianças selecionadas foram registrados seus dados gerais como peso, perímetro cefálico e presença de microcefalia ao nascer, além da idade, peso e perímetro cefálico no momento da aplicação da BTX-A e nível de comprometimento motor de acordo com a *Gross Motor Function Classification Measure* (GMFCS). Além disso, foram registradas informações a respeito da aplicação da BTX-A como data e musculaturas onde a medicação foi injetada.

Para avaliação dos efeitos da BTX-A foram consideradas dados de avaliações realizadas até 3 meses antes (pré-avaliação) e 4 semanas após (pós-avaliação) a aplicação dessa medicação. Dessas avaliações foram considerados dados referentes a avaliação do tônus muscular mensurado por meio da EAM 15 e mobilidade articular passiva mensurada por meio de medidas goniométricas. 16

A EAM foi utilizada para mensuração da resistência muscular ao movimento passivo, utilizada para avaliação do tônus muscular de pacientes com comprometimentos neurológicos como crianças com SCZ 2 e PC. 7 De acordo com essa escala, a resistência ao movimento passivo pode ser classificada entre 0 (sem aumento na tensão muscular) e 4 (segmento avaliado rígido sem movimentação). 17 No presente estudo, os grupos musculares dos membros superiores avaliados pela EAM foram: flexores e adutores do ombro, extensores e flexores do cotovelo, extensores e flexores do punho e flexores dos dedos. Já nos membros inferiores os grupos musculares avaliados foram os flexores do quadril, adutores do quadril, flexores e extensores do joelho, dorsiflexores e flexores plantares.

Para a avaliação da mobilidade articular passiva foi mensurada a máxima amplitude articular passiva com uso de um goniômetro manual. Para essa avaliação foram considerados os movimentos no plano sagital das articulações ombro, cotovelo, punho, quadril, joelho e tornozelo, assim como os movimentos de abdução das articulações do ombro e quadril no plano frontal.

Em todas as crianças, a aplicação de BTX-A foi realizada no ambulatório do Centro de Apoio a Criança com Microcefalia Dr. Arthur Eugênio Azevedo, com uso apenas de cloridrato de lidocaína em gel 20mg nas regiões da aplicações. O procedimento foi realizado sempre pelo mesmo profissional, levando em consideração o nível de hipertonia e as necessidades de cada criança. Para esse procedimento não foi utilizado qualquer instrumento auxiliar. Além disso, todas as avaliações antes e após a aplicação da BTX-A foram realizadas por profissionais treinados e com experiência no atendimento de crianças com SCZ e nos instrumentos de avaliação utilizados.

## Análise estatística

Para caracterização da amostra foi realizada a estatística descritiva, considerando a média e desvio padrão para variáveis contínuas como idade da criança e a máxima amplitude articular passiva avaliados antes e após a aplicação da BTX-A. Para variáveis categóricas, como resultados da EAM antes e após o procedimento, foram calculadas as frequências absoluta e relativa.

Em seguida, foi calculada a estatística inferencial. Para avaliação dos efeitos da BTX-A sobre a resistência muscular ao movimento passivo foram realizados testes de Wilcoxon pareado considerando como variáveis dependentes os resultados da EAM. Já para avaliação da BTX-A sobre a mobilidade articular foi inicialmente verificada a normalidade dos dados por meio do teste de Shapiro-Wilk. Diante da ausência de normalidade dos dados, essas variáveis também foram comparadas por meio do teste não paramétrico de Wilcoxon pareado. Todas as análises foram realizadas utilizando o software Medcalc (MedCalc Software BVBA, Ostend, Belgium), versão 19.0.7, tendo sido considerado o nível de significância estatística de 5%.

## Resultados

Ao todo foram registrados os dados de 13 crianças (9 meninos) das quais 7 (53,8%) apresentavam microcefalia ao nascer. No momento da aplicação da BTX-A as crianças tinham idade variando entre 70 e 89 (77 ± 7,1) meses, nenhuma era capaz de deambular de forma independente, 12 (92,3%) apresentavam tetraparesia espástica e todas apresentavam comprometimento motor grave sendo classificadas nos níveis IV (15,4%) e V (84,6%) de acordo com a GMFCS. A Tabela 1 apresenta os dados gerais individuais das crianças que fizeram parte do estudo.

**Tabela 1:** Perfil individual das crianças avaliadas e grupos musculares onde a BTX-A foi injetada

Participante	Idade na aplicação (meses)	Sexo	Perímetro cefálico (cm)		Peso (Kg)		Grupos musculares de aplicação da BTX-A
			Ao nascer	Na aplicação	Ao nascer	Na aplicação	
1	71	M	32,5	45	3,32	16,5	Biceps braquial, flexores de punho, flexores dos dedos e triceps sural
2	77	M	32	45	3,3	19,02	Biceps braquial, flexores de punho, flexores dos dedos e isquiostibiais
3	77	M	33	49	3,06	23,24	Biceps braquial, flexores de punho, adutores do quadril, isquiostibiais e triceps sural
4	70	F	28	41,5	3,01	17,83	Adutores do quadril e triceps sural
5	70	F	31,2	45,5	3,55	20,63	Biceps braquial, flexores dos dedos, isquiostibiais e triceps sural
6	76	M	31	49,5	2,02	22,86	Adutores do quadril e triceps sural
7	78	M	30	40	2,95	11,21	Biceps braquial, flexores de punho, adutores do quadril e isquiostibiais
8	87	M	30	45	2,78	17,94	Adutores do quadril, isquiostibiais e triceps sural
9*	76	F		41		16,59	Biceps braquial, flexores dos dedos, adutores do quadril e isquiostibiais
10	77	M	29	38,5	2,60	13,60	Flexores do punho e adutores do quadril
11	89	F	29,9	37,5	3,31	14,02	Adutores do quadril e isquiostibiais
12	88	M	29	45	2,64	17,83	Biceps braquial, flexores de punho e isquiostibiais
13	89	M	32	47	2,76	24,86	Flexores dos dedos e adutores do quadril e triceps sural

**Abreviações:** BTX-A, toxina botulínica-A; F, feminino; M, masculino. **Notas:** *Criança adotada sem informações sobre o nascimento.

A aplicação da BTX-A foi realizada nos membros superiores em 9 crianças, sendo os músculos com maior frequência de aplicação o bíceps braquial (n = 7) e flexor ulnar dos dedos (n = 6). Já nos membros inferiores, a aplicação foi realizada em todas as crianças em pelo menos um grupo muscular, sendo os com maior frequência de aplicação adutores do quadril (n = 9), isquiotibiais (n = 8) e tríceps sural (n = 7). Nenhum efeito adverso foi relatado pelos cuidadores entre as avaliações.

Após a aplicação, nenhuma mudança foi observada na máxima amplitude articular passiva para nenhum dos movimentos avaliados ( Tabela 2 ). Por outro lado, no que se refere ao tônus muscular, foram observadas diferenças significativas no grau de hipertonia dos grupos musculares flexores do cotovelo direito ( *p* = 0,01) e esquerdo ( *p* = 0,008), assim como dos abdutores de quadril direito ( *p* = 0,02) e esquerdo ( *p* = 0,04), conforme apresentado na Tabela 3 .

**Tabela 2:** Amplitude de movimento articular antes e após a aplicação da BTX-A

Movimento articular	Pré-aplicação			Pós-aplicação			
	Mediana	Mínimo	Máximo	Mediana	Mínimo	Máximo	Valor de *p*
**Flexão do ombro**							
Direito	170	90	180	168	90	180	0,93
Esquerdo	156	90	180	150	90	180	0,68
**Extensão de ombro**							
Direito ^a^	45	45	45	45	45	45	–
Esquerdo ^a^	45	45	45	45	45	45	–
**Abdução do ombro**							
Direito	160	90	180	150	110	180	0,68
Esquerdo	160	90	180	140	110	180	0,84
**Flexão de cotovelo**							
Direito ^a^	145	145	145	145	145	145	–
Esquerdo ^a^	145	145	145	145	145	145	–
**Extensão de cotovelo**							
Direito	0	0	120	0	0	60	0,12
Esquerdo	0	0	66	0	0	70	0,62
**Flexão de punho**							
Direito ^a^	90	30	140	90	30	70	–
Esquerdo	90	0	140	90	20	70	1,00
**Extensão de punho**							
Direito ^a^	70	0	70	70	20	70	–
Esquerdo	70	20	70	70	20	70	0,81
**Flexão de quadril**							
Direito	110	90	125	125	90	125	0,29
Esquerdo	110	90	125	120	90	125	0,62
**Extensão de quadril**							
Direito	10	0	10	10	0	10	0,62
Esquerdo	10	0	10	10	0	10	0,31
**Abdução de quadril**							
Direito	30	2	45	35	6	45	0,46
Esquerdo	24	0	45	30	8	45	–
**Adução de quadril**							
Direito ^a^	15	8	15	15	15	15	–
Esquerdo ^a^	15	15	15	15	15	15	–
**Flexão de joelho**							
Direito ^a^	140	140	140	140	140	140	–
Esquerdo ^a^	140	140	140	140	140	140	–
**Extensão de joelho**							
Direito	0	0	28	0	0	30	1,00
Esquerdo	10	0	135	0	0	40	0,06
**Dorsiflexão**							
Direito	20	10	30	0	0	20	0,81
Esquerdo ^a^	20	10	20	20	0	20	–
**Flexão plantar**							
Direito	45	30	45	45	35	45	0,21
Esquerdo	45	30	45	45	25	45	0,87

**Abreviação:** BTX-A, toxina botulínica-A.

**Nota:**^a^ Não foi possível realizar o teste estatístico pois não foram observadas mudanças numéricas no ângulo articular entre as avaliações.

## Discussão

A partir dos achados deste estudo foi evidenciado que a aplicação de BTX-A é capaz de promover a graduação do tônus de crianças com SCZ, apesar da não afetar a mobilidade articular. Além disso, nenhum efeito adverso foi registrado 4 semanas após a aplicação.

A hipertonia espástica caracteriza o tônus muscular da maioria das crianças com PC 18 e SCZ, 2 frequentemente relacionados a redução da mobilidade, contraturas e deformidades que podem comprometer o desenvolvimento e funcionalidade. 19 Especificamente em crianças com mobilidade reduzida, como aquelas classificadas nos níveis IV e V da GMFCS, a hipertonia generalizada também está relacionada a dor, desconforto e dificuldades nos cuidados cotidianos. 20 21 Assim, para essa população, tarefas simples como trocar de roupa podem ser dificultadas.

Apesar dos impactos do aumento do tônus muscular na saúde e qualidade da vida em crianças com comprometimentos motores graves e incapazes de deambular, estudos que avaliam os efeitos da BTX-A são escassos. Isso pode ser explicado pela necessidade de aplicação em múltiplos músculos e um maior risco de efeitos adversos. 7 Por outro lado, uma revisão sistemática proposta por Pin et al., 22 mostrou que o uso da BTX-A, quando aplicada em crianças classificadas nos níveis IV e V da GMFCS, se mostrou eficiente para redução da dor e facilitação de atividades cotidianas, além de promover melhoras nas habilidades motoras. Esses resultados, entretanto, devem ser vistos com cautela uma vez que a maioria dos estudos amostra apresentaram qualidade metodológica baixa ou moderada. 22

Em crianças com SCZ apenas dois estudos investigaram os efeitos da BTX-A. Um deles avaliou seus impactos no tratamento da sialorreia, 13 demonstrando a melhora na severidade dessa sintomatologia e a ocorrência de efeitos adversos em uma pequena porção da amostra (2/23). O outro estudo investigou os efeitos da BTX-A sobre a espasticidade e a performance motora em uma amostra que apresentava predominantemente com comprometimento motor grave (85%). Seus resultados demonstraram que a maioria dos pais relataram melhoras na amplitude de movimento ou na espasticidade de seus filhos após a aplicação da BTX-A, sem a ocorrência de nenhum efeito adverso. 14

Apesar da importância desses resultados, os estudos anteriores não demonstraram os impactos da aplicação da BTX-A sobre o tônus muscular de grupos musculares específicos avaliados por meios de escalas como a EAM. Isso porque Armani-Franceschi et al. 14 avaliou apenas a soma dos escores da EAM e não os grupos musculares individuais, o que limita a compreensão detalhada dos impactos da BTX-A em crianças com SCZ.

A avaliação da hipertonia de grupos musculares específicos pela EAM foi descrita no estudo transversal proposto por Tavares et al., 2 de acordo com o qual, a maioria das crianças com SCZ avaliadas apresentavam hipotonia axial e hipertonia apendicular, estando os flexores do cotovelo e adutores do quadril entre os grupos musculares que mais apresentam resistência ao movimento passivo. Esses grupos musculares estiveram entre os que mais frequentemente receberam a aplicação da BTX-A no presente estudos (53,8 e 69,2%, respectivamente), ao contrário do estudo de Armani-Franceschi et al. 14 no qual 50% das crianças receberam a aplicação no adutor longo e 35% no bíceps braquial.

Em crianças com comprometimentos neurológicos a hipertonia da musculatura do quadril é comum, relacionando-se à maior a susceptibilidade a luxações e dor. 19 Especificamente em crianças com SCZ, tem sido descrita uma alta prevalência de luxação e sublocação de quadril, fato que parece ter relação com o nível de hipertônia, 23 tendo como consequência dor e limitações funcionais. 24 Diante desses achados a redução da resistência ao movimento passivo após a aplicação da BTX-A, observada no presente estudo, pode representar alternativa terapêutica para preservação do quadril dessas crianças, alívio da dor e facilitação de seu manuseio nos cuidados cotidianos.

Até o presente momento nenhum estudo avaliou os impactos da hipertonia em crianças com SCZ. Apesar disso, a redução da resistência ao movimento passivo dos flexores de cotovelo, descritas no presente estudo podem representar um ponto positivo não apenas pela facilitação dos cuidados cotidianos, mas também pela facilitação do brincar e a interação da criança com o ambiente. Isso porque, em crianças com comprometimentos motores graves, a graduação do tônus muscular dos membros superiores pode facilitar tarefas de alcance e a manipulação de brinquedos. 7

No que se refere a mobilidade articular, mensurada a partir da máxima amplitude articular passiva, não foram observadas diferenças estatísticas. Algumas hipóteses podem ser levantadas para tal achado. Primeiro, o caráter multicausal da redução da amplitude de movimento articular, incluindo hipertônia e outros fatores, como encurtamento de músculos e tendões, alterações na matriz extracelular, dentre outros, podem comprometer a mobilidade articular em crianças com comprometimento neurológico. 25 26 27 Segundo, a necessidade de um maior tempo da associação entre BTX-A e programas de fisioterapia para que resultados terapêuticos significativos sejam atingidos. 28

Apesar da ausência de diferenças na amplitude de movimento articular antes e após a aplicação da BTX-A, os fisioterapeutas que acompanharam as crianças avaliadas durante o período do estudo relataram uma facilitação de sua manipulação durante as sessões de fisioterapia após o procedimento. Tal fato pode ser justificado pelos impactos da redução da resistência ao movimento passivo em segmentos corporais adjacentes. Apesar de não ser um desfecho do presente estudo, esse achado deve ser levado em consideração uma vez que a facilitação dos manuseios em programas de reabilitação pode resultar em melhores respostas terapêuticas a longo prazo.

Deve ser destacado os resultados aqui descritos podem ter sido influenciados pelo acompanhamento das crianças por uma equipe multidisciplinar que inclui fisioterapeutas. O número de sessões realizadas e as intervenções realizadas, entretanto, não foram controladas o que pode ser descrito como uma limitação do presente estudo. Outras limitações devem ser mencionadas, como o número reduzido de crianças bem avaliadas, a variedade de grupos musculares nos quais a BTX-A foi aplicada e o curto período de acompanhamento após o procedimento. Diante disso, sugere-se a realização de estudos futuros envolvendo um maior número de participantes e com o acompanhamento seriado e de maior duração a fim de verificar efeitos a longo prazo dessa aplicação.

## Conclusão

Apesar da hipertonia generalizada apresentada por crianças com SCZ, a aplicação da BTX-A no presente estudo se deu apenas em alguns grupos musculares, definidos de forma individualizada e a partir da avaliação clínica de uma equipe multidisciplinar especializada, a fim de evitar superdosagem. Isso pode explicar a ausência de efeitos adversos nas crianças investigadas, e sugerir a necessidade de um olhar clínico especializado e experiente para essa população.

O tratamento com BTX-A pode promover a graduação do tônus muscular de crianças com SCZ, com a redução da resistência ao movimento passivo. Essa terapêutica, entretanto, não foi capaz de promover mudanças na mobilidade articular, que é mensurada por meio da amplitude de movimento passiva.

**Tabela 3:** Avaliação do nível de espasticidade da amostra de acordo com a MAS antes e após a ampliação da BTX-A

Grupo muscular avaliado	MAS pré-aplicação, N (%)						MAS pós-aplicação, N (%)						
	0	1	1+	2	3	4	0	1	1+	2	3	4	Valor de *p*
**Adutores do ombro**													
Direita	5 (38,5)	1 (7,7)	2 (15,4)	3 (23,1)	2 (15,4)	0	4 (30,8)	6 (46,2)	0	3 (23,1)	0	0	0,15
Esquerda	6 (46,2)	2 (15,4)	1 (7,7)	2 (15,4)	2 (15,4)	0	3 (23,1)	8 (61,5)	0	2 (15,4)	0	0	0,54
**Flexores de quadril**													
Direita	7 (53,8)	3 (23,1)	0	3 (23,1)	0	0	9 (69,2)	4 (30,8)	0	0	0	0	0,09
Esquerda ^a^	7 (53,8)	3 (23,1)	0	3 (23,1)	0	0	8 (61,5)	3 (23,1)	0	2 (15,)	0	0	–
**Flexores de ombro**													
Direita	5 (38,5)	2 (15,4)	2 (15,4)	1 (7,7)	3 (23,1)	0	5 (38,5)	5 (38,5)	1 (7,7)	1 (7,7)	1 (7,7)	0	0,56
Esquerda	4 (30,8)	4 (30,8)	1 (7,7)	1 (7,7)	3 (23,1)	0	3 (23,1)	6 (46,2)	1 (7,7)	2 (15,4)	1 (7,7)	0	0,08
**Extensores do cotovelo**													
Direita	9 (75)	2 (16,7)	0	0	1 (8,3)	0	8 (61,5)	3 (23,1)	1 (7,7)	1 (7,7)	0	0	1,00
Esquerda	8 (66,7)	3 (25)	0	0	1 (8,3)	0	9 (69,2)	4 (30,8)	0	0	0	0	0,62
**Flexores de cotovelo**													
Direita	2 (15,4)	3 (23,1)	1 (7,7)	4 (30,8)	1 (7,7)	2 (15,4)	6 (46,2)	3 (23,1)	0	2 (15,4)	1 (7,7)	1 (7,7)	0,01
Esquerda	3 (23,1)	2 (154)	1 (7,7)	4 (30,8)	1 (7,7)	2 (15,4)	7 (53,8)	3 (23,1)	0	1 (7,7)	1 (7,7)	1 (7,7)	0,008
**Extensores do punho**													
Direita ^a^	8 (61,5)	3 (23,1)	1 (7,7)	1 (7,7)	0	0	10 (76,9)	2 (15,4)	0	1 (7,7)	0	0	–
Esquerda	8 (61,5)	3 (23,1)	0	2 (15,4)	0	0	10 (76,9)	2 (15,4)	0	0	1 (7,7)	0	0,87
**Flexores de punho**													
Direita	8 (61,5)	1 (7,7)	1 (7,7)	0	2 (15,4)	1 (7,7)	8 (61,5)	1 (7,7)	0	1 (7,7)	3 (23,1)	0	1,00
Esquerda	9 (69,2)	1 (7,7)	1 (7,7)	0	1 (7,7)	1 (7,7)	10 (76,9)	1 (7,7)	0	1 (7,7)	1 (7,7)	0	0,56
**Flexores dos dedos**													
Direita ^a^	9 (69,2)	2 (15,4)	0	1 (7,7)	1 (7,7)	0	10 (76,9)	2(15,4)	0	0	1 (7,7)	0	–
Esquerda	10 (76,9)	1 (7,7)	0	2 (15,4)	0	0	9 (69,2)	3 (23,1)	1 (7,7)	0	0	0	0,68
**Adutores do quadril**													
Direita	2 (15,4)	2 (15,4)	1 (7,7)	5 (38,5)	3 (23,1)	0	3 (23,1)	5(38,5)	2 (15,4)	3 (23,1)	0	0	0,02
Esquerda	2 (15,4)	2 (15,4)	1 (7,7)	5 (38,)	3 (23,1)	0	4 (30,8)	3 (23,1)	2 (15,4)	4 (30,8)	0	0	0,04
**Extensores do joelho**													
Direita	9 (69,2)	1 (7,7)	0	3 (23,1)	0	0	7 (53,8)	3 (23,1)	2 (15,4)	1 (7,7)	0	0	1,00
Esquerda	9 (69,2)	1 (7,7)	0	3 (23,1)	0	0	6 (46,2)	4 (30,8)	2 (15,4)	1 (7,7)	0	0	0,91
**Flexores de joelho**													
Direita ^a^	10 (76,9)	2 (15,4)	0	1 (7,7)	0	0	11 (84,6)	1 (7,7)	1 (7,7)	0	0	0	–
Esquerda	9 (69,2)	2 (15,4)	1 (7,7)	1 (7,7)	0	0	12 (92,3)	0	0	1 (7,7)	0	0	0,37
**Flexores plantares**													
Direita	2 (15,4)	5 (38,5)	2 (15,4)	2 (15,4)	2 (15,4)	0	4 (30,8)	2 (15,4)	0	5 (38,5)	0	2 (15,4)	0,82
Esquerda	1 (7,7)	5 (38,5)	3 (23,1)	2 (15,4)	2 (15,4)	0	3 (23,1)	1 (7,7)	0	7 (53,8)	2 (15,4)	0	0,49
**Dorsiflexores**													
Direita	6 (46,2)	3 (23,1)	2 (15,4)	0	2 (15,4)	0	8 (61,5)	3 (23,1)	0	1 (7,7)	0	1 (7,7)	0,64
Esquerda	6 (46,2)	3 (23,1)	2 (15,4)	0	2 (15,4)	0	9 (69,2)	1 (7,7)	0	3 (23,1)	0	0	0,57

**Abreviação:** MAS, escala de Ahworth modificada.

**Nota:**^a^ Não foi possível realizar o teste estatístico pois não foram observadas mudanças numéricas no ângulo articular entre as avaliações.
